# Diabetes Mellitus, Smoking Status, and Rate of Sputum Culture Conversion in Patients with Multidrug-Resistant Tuberculosis: A Cohort Study from the Country of Georgia

**DOI:** 10.1371/journal.pone.0094890

**Published:** 2014-04-15

**Authors:** Matthew J. Magee, Russell R. Kempker, Maia Kipiani, Nestani Tukvadze, Penelope P. Howards, K. M. Venkat Narayan, Henry M. Blumberg

**Affiliations:** 1 Department of Epidemiology, Rollins School of Public Health, Emory University, Atlanta, Georgia, United States of America; 2 Hubert Department of Global Health, Rollins School of Public Health, Emory University, Atlanta, Georgia, United States of America; 3 Division of Infectious Diseases, Department of Medicine, Emory University School of Medicine, Atlanta, Georgia, United States of America; 4 National Center for Tuberculosis and Lung Diseases, Tbilisi, Georgia; University of Minnesota, United States of America

## Abstract

**Introduction:**

Diabetes mellitus (DM) is a risk factor for active tuberculosis (TB) but little is known about the effect of DM on culture conversion among patients with multidrug-resistant (MDR)-TB. The primary aim was to estimate the association between DM and rate of TB sputum culture conversion. A secondary objective was to estimate the association between DM and the risk of poor treatment outcomes among patients with MDR-TB.

**Materials and Methods:**

A cohort of all adult patients starting MDR-TB treatment in the country of Georgia between 2009–2011 was followed during second-line TB therapy. Cox proportional models were used to estimate the adjusted hazard rate of sputum culture conversion. Log-binomial regression models were used to estimate the cumulative risk of poor TB treatment outcome.

**Results:**

Among 1,366 patients with sputum culture conversion information, 966 (70.7%) had culture conversion and the median time to conversion was 68 days (interquartile range 50–120). The rate of conversion was similar among patients with MDR-TB and DM (adjusted hazard ratio [aHR] 0.95, 95%CI 0.71–1.28) compared to patients with MDR-TB only. The rate of culture conversion was significantly less in patients that currently smoked (aHR 0.82, 95%CI 0.71–0.95), had low body mass index (aHR 0.71, 95%CI 0.59–0.84), second-line resistance (aHR 0.56, 95%CI 0.43–0.73), lung cavities (aHR 0.70, 95%CI 0.59–0.83) and with disseminated TB (aHR 0.75, 95%CI 0.62–0.90). The cumulative risk of poor treatment outcome was also similar among TB patients with and without DM (adjusted risk ratio [aRR] 1.03, 95%CI 0.93–1.14).

**Conclusions:**

In adjusted analyses, DM did not impact culture conversion rates in a clinically meaningful way but smoking did.

## Introduction

In the past 5 years, the relationship between type 2 diabetes mellitus (DM) and tuberculosis (TB) has emerged as a global public health priority. [1] Diabetes mellitus prevalence is increasing rapidly worldwide (currently estimated at 382 million and expected to reach 592 million by 2035 [2]), especially in low- and middle-income countries (LMIC) where TB is endemic. [3] Each year there are nearly 9 million new cases of TB, and an estimated 82% of cases occur in 22 high-burden countries all of which are LMIC. [4] The concern over co-occurring DM-TB epidemics is supported by literature reviews [5], [6], [7] and meta-analyses [8], [9] which estimate that patients with DM, compared to those without DM, have a 3-fold increased risk of developing active TB disease.

Global increases in multidrug-resistant (MDR)-TB (defined as TB strains resistant to at least isoniazid and rifampin) have brought additional challenges to TB control efforts. In 2013 the World Health Organization (WHO) reported that 3.6% of new cases and 20.2% of previously treated cases had MDR-TB, for estimated 450,000 new MDR-TB cases globally. [10] Second-line anti-TB drugs used in the treatment of MDR-TB are costly, and associated with a high rate of adverse effects. [11] In addition, MDR-TB treatment regimens must be extended for long periods of time (typically 20 months or longer), and the proportion of patients achieving treatment success is low (48% in 2012). [4] Consequently, management of MDR-TB treatment is difficult and requires national TB programs to use extensive financial and personnel resources. [12], [13].

Little is known about the relationship between DM and MDR-TB. Several studies have reported a high prevalence of DM among patients with MDR-TB, [14], [15], [16], [17] including an association between DM and MDR-TB after adjusting for confounding factors. [18] While DM has been associated with poor TB outcomes (including slower sputum culture conversion and higher risk of death and relapse [8]) among patients receiving first-line anti-TB therapy, whether DM affects outcomes among patients with MDR-TB is understudied. [19] The objective of this study was to estimate the association between DM and time to sputum culture conversion among adult pulmonary TB patients receiving MDR-TB second-line therapy. Secondarily, we aimed to 1) determine factors associated with culture conversion rates and 2) estimate the association between DM and the risk of poor treatment outcomes among patients with MDR-TB.

## Materials and Methods

A cohort of all adult (aged ≥18 years) patients with pulmonary MDR-TB starting second-line therapy between January 1, 2009 and December 30, 2011 in the country of Georgia was followed at the National Center for TB and Lung Disease (NCTBLD) in Tbilisi. Baseline data was collected on all patients who were then followed until a treatment outcome was achieved, or until January 2013, which ever occurred first. In Georgia, confirmation of pulmonary MDR-TB is defined by a positive sputum culture for *Mycobacterium tuberculosis* that is resistant to at least isoniazid (INH) and rifampicin (RIF). Second-line treatment at NCTBLD during the study period included initial empirical treatment that consisted of the following medications: capreomycin or kanamycin, levofloxacin, prothionamide, cycloserine, PAS and pyrazinamide. After final drug susceptibility test (DST) results became available, treatment regimens were individualized based on the results. When possible, regimens were designed to include at least four drugs to which the patient’s *M. tuberculosis* isolate was susceptible. All treatment regimens included a fluoroquinolone and an injectable agent for at least six months.

### Measures and Data Collection

The primary study outcome, *time to culture conversion*, was defined as time (in days) from MDR-TB treatment initiation until the first of two consecutive negative sputum cultures ≥30 days apart. Date of culture conversion was abstracted from patients’ medical records. Treatment outcomes were abstracted from the MDR-TB treatment registry and were based on WHO designated definitions. ^21,22^ The study’s secondary outcome, *poor treatment outcome*, was defined by combining the WHO outcome definitions of failed, defaulted, died, or transferred.

The primary study exposure of interest was diagnosed DM. Status of DM was assessed by physician diagnosis as recorded in the medical record. The secondary exposure of interest, current smoking status, was also assessed by physicians and recorded in patients’ medical records. Additional patient characteristics collected in the study included demographic and socio-behavioral information, concomitant infectious diseases, and TB clinical features. Body mass index (BMI) was calculated (kg/m^2^) from patient height and weight at the time of treatment initiation. Hepatitis co-infection status was classified as positive if the patient had documented positive serology for hepatitis A, B, or C viruses. Patients were screened for HIV co-infection by rapid test, positive tests were confirmed with Western Blot. [20] TB clinical features were abstracted from medical records documented at the time of MDR-TB treatment initiation and included patient information regarding previous TB treatment, chest radiographic findings (presence of any lung cavity, dissemination), smear results, and presence of extra-pulmonary TB.

Laboratory studies were performed at the Georgia National TB Reference Laboratory (NRL) in Tbilisi, which has received annual external quality assessment from Antwerp WHO Supranational TB Reference Laboratory since 2005. [21] Sputum smear AFB microscopy was performed using the Ziehl-Neelsen method and a standard semi-quantitative scale was used to classify the number of organisms present (negative through 4+). [22] Sputum cultures for *M. tuberculosis* were performed using Lowenstein-Jensen based solid medium, and the BACTEC MGIT 960 as previously described. [21] First-line DST was performed using the absolute concentration method or using the BACTEC, DST to second-line therapy was performed using the proportion method. [21] Patients with resistance to ofloxacin or either capreomycin or kanamycin were classified as having any second-line resistance.

### Data Analyses

All data were entered into an electronic database and analyzed using SAS 9.3 (SAS Institute Inc., Cary, NC USA). Bivariate associations were analyzed using χ2 tests for categorical variables, the Student’s t-test for normally distributed continuous variables (means), and the Kruskal-Wallis test for non-normally distributed continuous variables (medians). A two-sided p-value <0.05 was considered statistically significant for all analyses. Cox proportional hazards models were used to estimate hazard rate ratios (HR) and 95% confidence intervals (CI) for time to culture conversion. Patients were censored if at the time of treatment completion (for the outcomes completed, defaulted, died, or transferred) they did not have a prior documented sputum conversion. Proportional hazard assumptions were assessed graphically (log-negative-log curves), with goodness-of-fit (Schoenfeld residuals) and using time-dependent models. [23] Cumulative risk ratios (RR) and 95% CI for poor treatment outcome were modeled using log-binomial regression. Covariates considered to be known confounders were included in regression models based on significant bivariate associations with the primary exposure and outcomes, previous literature, or directed acyclic graph theory. [24].

### Ethics Statement

This study and was reviewed and approved by the Institutional Review Board of Emory University, Atlanta, USA. For the purposes of the study, all data were de-identified prior to access and analyses were performed anonymously by the researchers.

## Results

**Figure 1:**
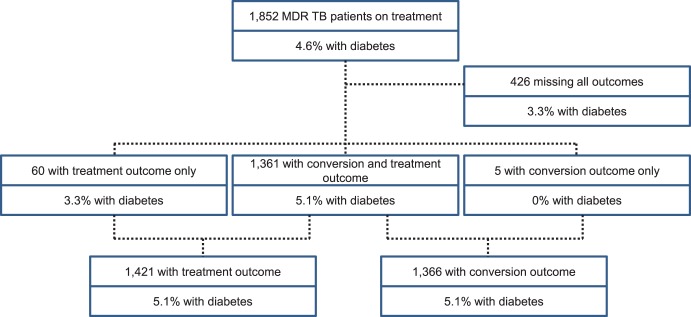
Study flow diagram of patients with MDR TB in Georgia, 2009–2011.

During the study period, 1,884 patients with laboratory confirmed pulmonary MDR-TB began second-line anti-TB therapy through the NCTBLD. Among these, 98.3% (1,852 of 1,884) were adults and included in the study. Culture conversion and censorship follow-up information was available for 73.8% (1,366 of 1,852) of patients and 76.7% (1,421 of 1,852) had a WHO defined treatment outcome (Figure 1). As of January 2013, an additional 426 MDR-TB patients remained on treatment, were missing culture conversion data, or had no treatment outcome information.

Of patients included in the study (N = 1,852), most were male (81.2%), current smoking was common (47.7%), and the median age was 35.1 years (Table 1). Diabetes mellitus was prevalent in 4.6% of patients with MDR-TB starting second-line therapy. Patients with MDR-TB and DM were significantly older (median age 48.8 vs. 34.6), less likely to have been imprisoned (20.2% vs. 40.5%), and heavier (median BMI 23.3 vs. 20.3) than MDR-TB patients without DM (p-value <0.05 for all comparisons). At the time of treatment initiation, the median fasting blood glucose (FBG) among patients with MDR-TB and DM was 124.3 mg/dl (IQR [interquartile range] 95.5–156.8) compared with 93.7 mg/dl (IQR 77.5–109.9) among patients without DM (p-value <0.05). HIV infection was prevalent in 4.5% of patients with MDR-TB patients without DM, but no patients with DM had HIV infection (p-value <0.05). Clinical TB characteristics at the time of second-line anti-TB drug treatment initiation were similar in patients with and without DM. Patients with DM were less likely to have received previous TB treatment (59.3% vs. 68.4%, p-value = 0.11) and more likely to have an AFB sputum smear grade ≥2 (55.3% vs. 44.0%, p-value <0.05) compared to MDR-TB patients without DM (Table 1).

**Table 1:** Diabetes mellitus and baseline characteristics of adult pulmonary MDR-TB patients in Georgia 2009–2012.

Patient characteristics at MDRTreatment start		No diabetes	Diabetes mellitus^A^	Total
		N = 1,766 (95.4)	N = 86 (4.6)	N = 1,852
		N (%)	N (%)	N (%)
Age (years)	18–34^B^	906 (51.3)	17 (19.8)	923 (49.8)
	35–44	423 (24.0)	20 (23.3)	443 (23.9)
	45–54	271 (15.4)	20 (23.3)	291 (15.7)
	≥55	166 (9.4)	29 (33.7)	195 (10.5)
Sex	Female	331 (18.7)	18 (20.9)	349 (18.8)
	Male	1,435 (81.3)	68 (79.1)	1,503 (81.2)
Ever imprisoned^B^	No	1,030 (59.5)	67 (79.8)	1,097 (60.5)
	Yes	700 (40.5)	17 (20.2)	717 (39.5)
Current smoker	No	916 (51.9)	53 (61.6)	969 (52.3)
	Yes	850 (48.1)	33 (38.4)	883 (47.7)
Alcohol use	None	1,079 (64.7)	50 (60.2)	1,129 (65.3)
	Moderate	468 (28.5)	30 (36.1)	498 (28.8)
	Heavy	98 (6.0)	3 (3.6)	101 (5.8)
Body mass index	<18.5^B^	391 (23.8)	4 (4.9)	395 (22.9)
	18.5–24.9	1,142 (69.5)	52 (63.4)	1,194 (69.1)
	25–29.9	96 (5.8)	23 (28.1)	119 (6.9)
	≥30	15 (0.9)	3 (3.7)	18 (1.0)
	Missing^C^	122	4	126
RBG, mg/dl	Mean (STD)	95.5 (25.2)	118.9 (46.8)	97.3 (27.0)
	Median (IQR)^B^	93.7 (77.5–109.9)	124.3 (95.5–156.8)	93.7 (79.3–111.7)
	Missing	859	58	917
HIV status^B^	Negative	1,420 (95.5)	67 (100.0)	1,487 (95.7)
	Positive	67 (4.5)	0	67 (4.3)
	Missing	279	19	298
Hepatitis (A, B, or C)	Negative	1,592 (90.2)	81 (94.2)	1,673 (90.3)
	Positive	174 (9.9)	5 (5.8)	179 (9.7)
Previous TB (retreatment)	No	575 (32.6)	35 (40.7)	610 (32.9)
	Yes	1,191 (67.4)	51 (59.3)	1,242 (67.1)
Any second-line drugresistance	No	1,537 (88.2)	73 (88.0)	1610 (88.2)
	Yes	205 (11.8)	10 (12.0)	215 (11.8)
AFB smear grade^B^	Negative	445 (26.1)	6 (7.1)	451 (25.2)
	1+	508 (29.8)	32 (37.7)	540 (30.2)
	2+	281 (16.5)	20 (23.5)	301 (16.8)
	3+	242 (14.2)	12 (14.1)	254 (14.2)
	4+	227 (13.3)	15 (17.7)	242 (13.5)
Any lung cavity	No	1,179 (73.1)	63 (75.0)	1,242 (73.2)
	Yes	434 (26.9)	21 (25.0)	455 (26.8)
	Missing	153	2	155
Disseminated TB^D^	No	1,342 (83.2)	69 (82.1)	1,411 (83.5)
	Yes	271 (16.8)	15 (17.9)	286 (16.9)
	Missing	153	2	155
Extra-pulmonary^D^	No	1,661 (94.1)	83 (96.5)	1,744 (94.2)
	Yes	105 (5.9)	3 (3.5)	108 (5.8)

Table 1 Abbreviations: RBG-random blood glucose; MDR-multidrug-resistant; STD-standard deviation; IQR-interquartile range; AFB-acid fast bacilli.

A Based on medical records or self-reported by MDR-TB patients.

B Statistically significant, two-sided p-value <0.05.

C Variables with missing >5% among patients were reported in the table as a separate category but not calculated within the frequency distribution.

D All patients had pulmonary MDR-TB; extra-pulmonary/disseminated includes those with both pulmonary and extra-pulmonary/disseminated.

Among patients with MDR-TB included in the study, 70.7% (966 of 1,366) converted sputum cultures from positive to negative during second-line therapy (Table 2). The median time to culture conversion from positive to negative was 64 (IQR 58–106) days among patients with MDR-TB and DM compared to 69 (IQR 48–118) days among patients with MDR-TB only (p = 0.86). In unadjusted analysis, the proportion of patients with MDR-TB and DM who converted sputum cultures during treatment was similar compared to patients without DM (RR 1.08, 95% CI 0.94–1.23). After adjusting for confounding covariates in a Cox model, the estimated rate of converting to negative was modestly but non-significantly lower in patients with MDR-TB and DM (adjusted hazard ratio [aHR] 0.93, 95%CI 0.71–1.23) compared to MDR-TB patients without DM. In the same multivariable model, other characteristics significantly associated with lower hazards of culture conversion included smoking (aHR 0.82, 95%CI 0.71–0.95), BMI <18.5 (aHR 0.71, 95%CI 0.59–0.84), lung cavity on chest radiograph (aHR 0.70, 95%CI 0.59–0.83), and disseminated TB (aHR 0.75, 95%CI 0.62–0.90). Drug resistance had the greatest estimated effect on conversion time, patients with any second-line resistance (aHR 0.56, 95%CI 0.43–0.73), had nearly half the hazard of conversion compared to patients with second-line susceptible MDR-TB.

**Table 2:** Bivariate and multivariable hazard rate ratios for patient characteristics associated with sputum culture conversion among patients with MDR-TB in Georgia, 2009–2011.

Patient characteristicat MDR TB treatmentstart		Converted	Median	cHR	aHR^B^
		966/1366 (70.7)	Conversion		
		N/Total (%)	(IQR)^A^	(95% CI)	(95% CI)
Diabetes	No	913/1,296 (70.5)	69 (48–119)	1.00	1.00
	Yes	53/70 (75.7)	64 (58–106)	1.12 (0.85, 1.48)	0.95 (0.71, 1.28)
Age (years)	18–34	506/676 (74.9)	68 (46–117)	1.00	1.00
	35–44	215/330 (65.2)	71 (41–119)	**0.81 (0.69, 0.95)**	0.86 (0.73, 1.02)
	45–54	138/211 (65.4)	75 (58–157)	**0.80 (0.66, 0.96**)	0.93 (0.76, 1.13)
	≥55	107/149 (71.8)	65 (50–101)	1.07 (0.87, 1.32)	1.10 (0.88, 1.37)
Sex	Female	200/272 (73.5)	65 (40–99)	1.00	1.00
	Male	766/1,094 (70.0)	70 (49–121)	0.86 (0.73, 1.00)	0.98 (0.82, 1.18)
Ever imprisoned	No	634/876 (72.4)	67 (55–112)	1.00	1.00
	Yes	332/490 (67.8)	72 (44–123)	**0.85 (0.75, 0.97)**	1.00 (0.86, 1.17)
Current smoker	No	554/734 (75.5)	65 (49–111)	1.00	1.00
	Yes	412/632 (65.2)	75 (48–123)	**0.77 (0.68, 0.88)**	**0.82 (0.71, 0.95)**
Alcohol use	None	679/935 (72.6)	66 (41–106)	1.00	1.00
	Moderate	244/351 (69.5)	78 (56–127)	0.86 (0.77, 1.02)	1.01 (0.86, 1.19)
	Heavy	43/80 (53.8)	91 (61–141)	**0.62 (0.46, 0.83)**	0.79 (0.56, 1.09)
Body mass index	<18.5	161/289 (55.7)	82 (60–125)	**0.64 (0.54, 0.76)**	**0.71 (0.59, 0.84)**
	18.5–24.9	641/872 (73.5)	66 (41–111)	1.00	1.00
	≥25	90/105 (85.7)	67 (41–121)	**1.27 (1.02, 1.59)**	**1.29 (1.03, 1.63)**
	Missing	74/100 (74.0)	74 (59–120)		
RBG, mmol/l	<7.7	411/556 (73.9)	64 (39–109)	1.00	
	≥7.7	33/47 (70.2)	95 (57–138)	0.85 (0.60, 1.22)	
	Missing	522/763 (68.4)	76 (56–121)		
HIV status	Negative	772/1,079 (71.6)	67 (48–113)	1.00	
	Positive	28/49 (57.1)	76 (58–132)	0.76 (0.52, 1.11)	
	Missing	166/238 (69.8)	81 (50–123)		
Hepatitis (A, B, or C)	Negative	877/1,228 (71.4)	69 (52–118)	1.00	
	Positive	89/138 (64.5)	62 (38–121)	0.91 (0.73, 1.13)	
Previous TB retreatment	No	340/433 (78.5)	69 (56–104)	1.00	1.00
	Yes	626/933 (67.1)	69 (46–122)	**0.78 (0.68, 0.89)**	0.96 (0.83, 1.12)
Any 2nd line resistance^C^	No	903/1,214 (74.4)	69 (47–117)	1.00	1.00
	Yes	63/152 (41.5)	87 (56–127)	**0.46 (0.36, 0.60)**	**0.56 (0.43, 0.73)**
AFB smear grade^D^	Negative	269/373 (72.1)	61 (33–75)	1.00	
	1+	300/409 (73.4)	67 (50–109)	0.86 (0.73, 1.01)	
	2+	161/216 (74.5)	78 (57–123)	0.84 (0.69, 1.03)	
	3+	133/193 (68.9)	92 (63–125)	**0.70 (0.57, 0.86)**	
	4+	103/175 (58.9)	105 (71–154)	**0.52 (0.42, 0.66)**	
AFB smear grade^D^	Neg/1–2+	730/998 (73.2)	63 (37–100)	1.00	
	3–4+	236/368 (64.1)	95 (66–132)	**0.68 (0.59, 0.79)**	
Any lung cavity^E^	No	752/1,006 (74.8)	63 (37–100)	1.00	1.00
	Yes	214/360 (59.4)	95 (66–132)	**0.63 (0.54, 0.73)**	**0.70 (0.59, 0.83)**
Dissem TB	No	818/1,138 (71.9)	69 (52–119)	1.00	1.00
	Yes	148/228 (64.9)	70 (35–118)	**0.84 (0.70, 0.99)**	**0.75 (0.62, 0.90)**
Extra-pulmonary^F^	No	921/1,282 (71.8)	69 (48–118)	1.00	1.00
	Yes	45/84 (53.6)	76 (49–103)	0.87 (0.75, 1.01)	0.90 (0.77, 1.06)

Table 2 Abbreviations: IQR-interquartile range; MDR-multidrug-resistant; cHR-crude hazard rate ratio; aHR-adjusted hazard rate ratio; RBG-random blood glucose; AFB-acid fast bacilli; Neg-negative; Dissem-disseminated TB; **Bold** indicates statistically significant, two sided p-value <0.05.

A Among patients who converted, median time (days) from MDR TB treatment initiation until first of two consecutive negative sputum cultures ≥30 days apart among patients.

B The adjusted model included all variables with estimates in the aHR column.

C Any resistance to a second-line TB drug.

D Violated proportional hazards assumptions and not included in adjusted model.

E Missing data for this variable was recoded into no/null category.

F All patients were pulmonary, extra-pulmonary includes those with both pulmonary and extra-pulmonary TB.

Overall 50.6% (719 of 1,421) of patients had a treatment outcome defined as cured or completed (Table 3). After adjusting for multiple confounders in a log-binomial model, risk of poor treatment outcome did not significantly differ among those with or without DM (adjusted risk ratio [aRR] 1.03, 95%CI 0.93–1.14) (Table 4). However, cumulative risk of poor treatment outcome was significantly associated with BMI <18.5, any second-line resistance, and AFB smear grade (4+). Failure to convert sputum culture to negative was most strongly associated with poor treatment outcome (aRR 1.27, 95%CI 1.20–1.35) including increased risk of death (RR 7.89, 95%CI 5.26–11.85) (data not shown).

**Table 3:** Diabetes mellitus and treatment outcomes among adult pulmonary MDR-TB patients in Georgia 2009–2012.

WHO definedtreatment outcome	No diabetes	Diabetes^A^	Risk Ratio^B^
	N = 1,349 (94.9)	N = 72 (5.1)	(95% CI)
	N/Total (%)	N/Total (%)	
*Favorable outcome*	*683/1,349 (50.6)*	*36/72 (50.0)*	*0.99 (0.78, 1.25)*
Cured	406 (30.1)	26 (36.1)	1.20 (0.87, 1.65)
Completed	277 (20.5)	10 (13.9)	0.68 (0.38, 1.21)
*Poor Outcome*	*666/1,349 (49.4)*	*36/72 (50.0)*	1.01 (0.80, 1.28)
Failed	61 (4.5)	3 (4.2)	0.92 (0.30, 2.87)
Defaulted	465 (34.5)	29 (40.3)	1.17 (0.87, 1.56)
Died	129 (9.6)	3 (4.2)	0.44 (0.14, 1.34)
Transferred	11 (0.8)	1 (1.4)	1.70 (0.22, 13.01)

Table 3 Abbreviations: WHO-World health organization; RR-risk ratio; CI-confidence interval.

A Physician diagnosed diabetes mellitus.

B No diabetes mellitus was considered as the referent group.

**Table 4:** Bivariate and multivariable analyses of patient characteristics associated with cumulative risk of poor treatment outcome among adult pulmonary MDR-TB patients in Georgia 2009–2011.

Patient characteristicat MDR TB treatmentstart		Poor Outcome^A^	cRR	aRR^B^
		702/1,421 (44.3)		
		N/Total (%)	(95% CI)	(95% CI)
Diabetes	No	666/1,349 (49.4)	1.00	1.00
	Yes	36/72 (50.0)	1.01 (0.80, 1.28)	1.03 (0.93, 1.14)
Age (years)	18–34	332/708 (46.9)	1.00	1.00
	35–44	170/337 (50.5)	1.08 (0.94, 1.23)	0.98 (0.93, 1.04)
	45–54	126/224 (56.3)	**1.20 (1.04, 1.38)**	1.01 (0.94, 1.09)
	≥55	74/152 (48.7)	1.04 (0.87, 1.24)	1.00 (0.93, 1.09)
Sex	Female	102/283 (36.0)	1.00	1.00
	Male	600/1,138 (52.7)	**1.46 (1.24, 1.72)**	0.95 (0.88, 1.02)
Ever imprisoned	No	399/910 (43.9)	1.00	1.00
	Yes	303/511 (59.3)	**1.35 (1.22, 1.50)**	1.03 (0.97, 1.09)
Current smoker	No	329/763 (43.1)	1.00	1.00
	Yes	373/658 (56.7)	**1.31 (1.18, 1.46)**	1.03 (0.98, 1.09)
Alcohol use	None	451/967 (46.6)	1.00	1.00
	Moderate	194/368 (52.7)	**1.13 (1.01, 1.27)**	1.00 (0.94, 1.06)
	Heavy	57/86 (66.3)	**1.42 (1.20, 1.68)**	1.05 (0.95, 1.15)
Body mass index	<18.5	190/302 (62.9)	**1.33 (1.19, 1.49)**	**1.07 (1.01, 1.13)**
	18.5–24.9	429/910 (47.1)	1.00	1.00
	≥25	42/105 (40.0)	0.85 (0.66, 1.08)	0.96 (0.87, 1.13)
	Missing	41/104 (39.4)		
RBG, mmol/l	<7.7	289/576 (50.2)	1.00	
	≥7.7	23/46 (50.0)	1.00 (0.73, 1.35)	
	Missing	390/799 (48.8)		
HIV status	Negative	549/1,114 (49.3)	1.00	1.00
	Positive	34/52 (65.4)	**1.33 (1.08, 1.63)**	1.06 (0.95, 1.17)
	Missing	119/255 (46.7)		
Hepatitis (A, B, or C)	Negative	619/1,282 (48.3)	1.00	1.00
	Positive	83/139 (59.7)	**1.24 (1.07, 1.43)**	1.01 (0.93, 1.09)
Previous TB retreatment	No	181/447 (40.5)	1.00	1.00
	Yes	521/974 (53.5)	**1.32 (1.17, 1.50)**	1.10 (0.96, 1.25)
Any 2nd line Resistance^C^	No	589/1,263 (46.6)	1.00	1.00
	Yes	113/158 (71.5)	**1.53 (1.36, 1.72)**	**1.09 (1.01, 1.17)**
AFB smear grade^D^	Negative	155/408 (38.0)	1.00	1.00
	1+	214/415 (51.6)	**1.36 (1.16, 1.59)**	1.05 (0.99, 1.12)
	2+	115/223 (51.6)	**1.36 (1.14, 1.62)**	1.04 (0.97, 1.12)
	3+	106/196 (54.1)	**1.42 (1.19, 1.70)**	1.03 (0.96, 1.12)
	4+	112/179 (62.6)	**1.65 (1.39, 1.95)**	**1.09 (1.01, 1.19)**
Any lung Cavity^C^	No	486/1,051 (46.2)	1.00	1.00
	Yes	216/370 (58.4)	**1.26 (1.13, 1.41)**	1.02 (0.97, 1.08)
Disseminated TB^C^	No	574/1,187 (48.4)	1.00	
	Yes	128/234 (54.7)	1.13 (0.99, 1.29)	
Extra-Pulmonary^D^	No	655/1,333 (49.1)	1.00	
	Yes	47/88 (53.4)	1.09 (0.89, 1.33)	
Fail culture convert	No	328/961 (34.1)	1.00	1.00
	Yes	346/400 (86.5)	**2.54 (2.30, 2.79)**	**1.27 (1.20, 1.35)** E
Culture conversiontime Days^F^	≤48	82/242 (33.9)	1.00	
	49–118	143/483 (29.6)	0.87 (0.70, 1.09)	
	≥119	103/236 (43.6)	**1.29 (1.03, 1.62)**	
	No convert	346/400 (86.5)	**2.55 (2.13, 3.06)**	

Table 4 . Abbreviations: MDR-multidrug-resistant; cRR-crude risk ratio; aRR-adjusted risk ratio; AFB-acid fast bacilli; **Bold** indicates statistically significant, two sided p-value <0.05.

A Poor treatment outcome was defined as failed, defaulted, died, or transferred.

B Variables in the adjusted model included all those with reported aRR estimates except *Failure to convert culture*.

C Missing values were coded as no, none, or negative.

D All patients were pulmonary, extra-pulmonary includes those with both pulmonary and extra-pulmonary TB.

E Adjusted estimate obtained from a separate model for aRR due to 60 patients with treatment outcome who were missing culture conversion status.

F Categories created by tertiles of conversion time.

## Discussion

This cohort study of adult pulmonary MDR-TB patients from the country of Georgia found that after adjusting for important confounding factors, the rate of sputum culture conversion and the risk of poor treatment outcome was similar in MDR-TB patients with and without DM. Importantly, in multivariable analyses we did find factors significantly associated with lower conversion rates, including current smoking, cavitary disease, and disseminated TB. We also found low BMI (<18.5) and any second-line drug resistance were significantly associated with both lower culture conversion rates and greater risk of poor treatment outcome. Compared to patients with MDR-TB who culture converted, failure to convert sputum culture to negative was strongly associated with poor treatment outcome and increased the risk of death by nearly eight-fold.

Few other studies have examined the effect of DM status on time until sputum culture conversion or risk of poor TB treatment outcome among patients with MDR-TB. Consistent with our adjusted HR estimate (0.95, 95% CI 0.71–1.28), a 2012 study of MDR-TB patients from five countries also reported a lower but not significant *unadjusted* rate (HR 0.76, 95% CI 0.54–1.06) of sputum culture conversion among patients with DM. [25] Unlike our findings in patients with MDR-TB, previous studies among patients with drug sensitive TB have found that the presence of DM delays culture conversion time. [26] For example, a study published in 2013 from Mexico reported the proportion of TB patients on first line TB therapy who converted sputum cultures to negative ≥60 days of treatment was significantly greater in patients with DM (45.9%) compared to those without DM (37.2%). [27] Earlier studies have also reported an association between cavitary disease, [25], [28] and second-line drug resistance [29] with lower rates of (or longer time to) culture conversion in patients with MDR-TB. Like our results, low BMI (<18.5), second-line drug resistance, higher baseline AFB smear grade, and failure to convert sputum cultures are consistently reported predictors of poor MDR-TB treatment outcomes. [30], [31].

Existing evidence strongly suggests that smoking is risk factor for developing active TB [32] and is associated with TB treatment failure. [33] Corroborating the association between smoking and TB, we also found that among patients with MDR-TB, current smoking was a risk factor for lower rates of sputum culture conversion. After controlling for confounding factors, smoking was significantly associated with a 20% reduction in the rate of conversion. Our finding is consistent with a prior Brazilian study, which found that smokers had an increased odds (2.3) of being culture positive after two months of TB first-line treatment compared to never smokers. [34] Because patients with MDR-TB who also currently smoke have lower rates of sputum culture conversion, they may also be infectious for longer periods of time which could lead to additional transmission of MDR-TB. Consequently, smoking cessation programs designed for TB patients potentially could not only improve individual outcomes but also indirectly reduce both community and nosocomial transmission of MDR-TB.

This study was subject to limitations. First, DM status was not systematically measured for all patients at the time of second-line TB treatment initiation, which may have resulted in misclassification or an underestimation of the prevalence of dysglycemia among those with MDR-TB. However, patients with MDR-TB previously diagnosed with DM or who were taking drugs for glycemic control likely had DM status documented in their medical records and consequently the specificity of our measured DM status was likely high. We do not have reason to believe that misclassification of DM status was differential with respect to sputum culture conversion, and therefore our estimated effect of the exposure (DM) on the outcome (culture conversion) would plausibly be expected to be biased toward the null. [35] Consequently, our estimated HR for the effect of DM on culture conversion may be lower than if DM status was measured in all patients without any error. Second, the measurement of DM in this study did not include a comprehensive assessment of glucose control (e.g., hemoglobin A1c) and therefore we were unable to determine the effect of DM management on sputum culture conversion. Third, 26.2% of patients who began MDR-TB treatment did not have culture conversion information available at the end of the study and as a result, the generalizability of the findings to all MDR-TB patients in Georgia may be limited. Missing treatment outcome information for MDR-TB patients is high in most national TB programs–globally, 28% of MDR-TB patients on second-line therapy were lost to follow up or did not have treatment outcome information reported in 2011. [4] Nonetheless, data from our analyses were collected from nearly all diagnosed MDR-TB patients in Georgia during a three-year period and the population-based nature of the information is a key study strength.

### Conclusions

Few other studies have examined the effect of DM on time to sputum culture conversion in drug-susceptible TB cohorts, and to our knowledge we present the first analysis of the adjusted effect of DM on culture conversion among TB patients with confirmed MDR. In addition, our study was able to control for multiple confounding factors not accounted for in previous studies of culture conversion and poor treatment outcome in patients with MDR-TB.

Although previous studies suggest DM may increase the time to sputum culture conversion and risk of treatment failure among drug susceptible TB patients, our results did not detect a clinically meaningful difference in culture conversion rates or risk of poor treatment outcome in patients with MDR-TB from the country of Georgia. We did identify important predictive factors for both lower culture conversion rates and poor treatment outcomes including current smoking, low BMI, second-line drug resistance, cavitary disease, disseminated TB, and AFB smear grade.
